# Draft Genome Sequence of *Chloroflexus* sp. Strain isl-2, a Thermophilic Filamentous Anoxygenic Phototrophic Bacterium Isolated from the Strokkur Geyser, Iceland

**DOI:** 10.1128/genomeA.00714-16

**Published:** 2016-07-21

**Authors:** Vasil A. Gaisin, Timophey M. Ivanov, Boris B. Kuznetsov, Vladimir M. Gorlenko, Denis S. Grouzdev

**Affiliations:** Research Center of Biotechnology of the Russian Academy of Sciences, Moscow, Russian Federation

## Abstract

We report here the draft genome sequence of the thermophilic filamentous anoxygenic phototrophic bacterium *Chloroflexus* sp. strain isl-2, which was isolated from the Strokkur geyser, Iceland, and contains 5,222,563 bp with a G+C content of 59.65%. The annotated genome sequence offers the genetic basis for understanding the strain’s ecological role as a phototrophic bacterium within the bacterial community.

## GENOME ANNOUNCEMENT

*Chloroflexus* sp. strain isl-2 is a thermophilic filamentous anoxygenic phototrophic bacterium (FAPB) of the family *Chloroflexaceae* of the phylum *Chloroflexi*. Strain isl-2 was isolated from a water-sand suspension sampled from the Strokkur geyser basin, Iceland. Previously, *Chloroflexus*-like bacteria in the Strokkur geyser were described as participants in the biomineralization process; however, information about the biological properties of these bacteria was limited (1, 2). The genome sequence of strain isl-2 is expected to provide new insights into the potential ecological role of FAPB in the Strokkur ecosystem.

Genomic DNA was extracted according to Wilson (3), with minor modifications. The DNA was sonicated on a Covaris S2 device to the average insert size of 250 bp. A sequence library was constructed with the NEBNext DNA library prep reagent set for Illumina according to the manufacturer’s protocol. The library was paired-end sequenced using the HiSeq1500 platform with 150-bp read lengths for both reads.

Approximately 2.82 million reads were generated, providing 30-fold genome coverage. The obtained reads were assembled using SPAdes version 3.1.0 (4). The resulting 125 contigs were submitted to the ProDeGe website (5) for automatic decontamination. Finally, the assembled sequence was submitted to the NCBI Prokaryotic Genome Annotation Pipeline (PGAP) (6) for annotation*.*

The draft genome sequence of *Chloroflexus* sp. isl-2 revealed a genome size of 5,222,563 bp with an average G+C content of 59.65%, which was close to the G+C contents of *Chloroflexus aurantiacus* J-10-fl^T^ (56.70%) and *Chloroflexus aggregans* DSM 9485^T^ (56.43%). The draft genome contained a total of 3,979 open reading frames, including at least 49 tRNAs, eight rRNAs, and two ncRNAs. The 16Sr RNA gene sequence was 96.6% and 97.0% similar to those of the type strains *C. aurantiacus* J-10-fl^T^ (CP000909) and *C. aggregans* DSM 9485^T^ (CP001337), respectively.

The genomic sequence includes genes encoding elements of photosynthetic apparatus that are typical for *Chloroflexus* spp. (namely, quinone-type photosynthetic reaction centers, a light-harvesting complex, chlorosome proteins, and enzymes required for the synthesis of bacteriochlorophylls *a* and *c*). The genes for the enzymes of the 3-hydroxypropionate cycle are present, but photoautotrophic growth was not observed.

### Nucleotide sequence accession numbers.

This whole-genome shotgun project has been deposited at DDBJ/ENA/GenBank under the accession number LWQS00000000. The version described in this paper is the first version, LWQS01000000.
